# Partnership for a Healthier America: Creating Change Through Private Sector Partnerships

**DOI:** 10.1007/s13679-017-0253-z

**Published:** 2017-04-24

**Authors:** Caitlin Simon, S. Lawrence Kocot, William H. Dietz

**Affiliations:** 1 Partnership for a Healthier America, 2001 Pennsylvania Avenue NW, 9th Floor, Washington, DC 20006 USA; 2 Center for Healthcare Regulatory Insight, KPMG, LLP, Washington, DC USA; 3 Sumner M. Redstone Center, Milken Institute School of Public Health, Washington, DC USA

**Keywords:** Nutrition, Physical activity, Childhood obesity, Business, Partnership for a Healthier America, *Let’s Move!*

## Abstract

**Purpose of Review:**

This review provides background on the formation of the Partnership for a Healthier America (PHA), that was created in conjunction with the *Let’s Move!* initiative, and an overview of its work to date.

**Recent Findings:**

To encourage industry to offer and promote healthier options, PHA partners with the private sector. Principles that guide PHA partnerships include ensuring that partnerships represent meaningful change, partners sign a legally binding contract and progress is monitored and publicly reported.

**Summary:**

Since 2010, PHA has established private sector partnerships in an effort to transform the marketplace to ensure that every child has the chance to grow up at a healthy weight. Many agreements between PHA and its industry partners align with the White House Task Force Report on Childhood Obesity. The reach and impact of over 200 partnerships attest to the success of this initiative.

## Introduction

In the late 1990s and early 2000s, voluntary agreements emerged as a promising strategy to engage companies around health issues. Private-public partnerships were forming to solve some of the most critical global public health issues, and both the World Bank and World Health Organization promoted these approaches. When the Clinton Foundation and the American Heart Association founded the Alliance for a Healthier Generation (AHG) in 2005, two of their four fundamental “pillars” were voluntary agreement approaches with food and beverage companies and with health insurers.

In 2009, First Lady Michelle Obama was considering a major initiative to battle childhood obesity. The *Let’s Move!* initiative would include a broad set of activities; one strategy included work with industry. In late 2009, discussions between a number of organizations, including the Robert Wood Johnson Foundation (RWJF), AHG and the White House, led to the identification of a need for a national organization that could drive voluntary changes by business to address childhood obesity with public-private partnerships, and hold companies accountable to implement those changes. Additionally, these discussions acknowledged that ending our nation’s childhood obesity crisis required a long-term “generational” strategy and therefore needed an organization that would exist beyond the timeframe of the Administration to continue efforts initiated by Mrs. Obama. To meet these needs, the Partnership for a Healthier America (PHA) was created.

### Creation of PHA and Early Work

As shown in Table 1, The *Let’s Move!* initiative was formally launched on February 9, 2010. This launch by the First Lady also included the announcement of a new independent foundation, The Partnership for a Healthier America, to “accelerate existing efforts addressing childhood obesity and facilitate new commitments towards the national goal of solving childhood obesity within a generation” [1]. President Barack Obama kicked off the launch by signing a Presidential Memorandum creating a White House Task Force on Childhood Obesity, with a mandate to develop a national action plan within 90 days to maximize federal resources and set concrete benchmarks towards the first lady’s national goal.

**Table 1:** Partnership for a Healthier America’s Key Dates

Year	Activities
2010	February: *Let’s Move!* and PHA launched March: 1st PHA Board Meeting May: Publication of the White House Taskforce Report on Childhood Obesity; HWCF commitment
2011	January: 1st full-time PHA staff hired June: Let’s Move Childcare launched and Bright Horizons (PHA’s 1st Childcare) commitment July: 1st food access commitment November: PHA’s 1st Building a Healthier Future Summit
2012	September: Healthier Hospital Food Initiative launched with 17 hospital systems
2013	February: Let’s Move Active Schools launch March: PHA’s 2nd Building a Healthier Future Summit; release of PHA’s first progress report (for 2012) September: Drink UP launch
2014	March: PHA’s 3rd Building a Healthier Future Summit; release of PHA’s 2013 Progress Report; announcement of PHA’s 1st convenience store commitment November: Healthier Campus Initiative launched with 20 campus partners
2015	February: PHA’s 4th Building a Healthier Future Summit; Announcement of PHA’s first active design commitment; FNV announcement May: Release of PHA’s 2014 Progress Report June: FNV launches in lead markets
2016	May: PHA’s 5th Building a Healthier Future Summit; release of PHA’s 2015 Progress Report

PHA was incorporated on January 13, 2010, and its board of directors was seated on March 23, 2010. In addition, on that date, the board appointed a Founders Committee to serve in an advisory capacity to the Board of Directors. Members of the Committee included the leaders of The California Endowment, Kaiser Permanente, Nemours, Robert Wood Johnson Foundation and W.K. Kellogg Foundation. These foundations and organizations had already worked together for several years to address the challenge of obesity.

On April 1, 2010, the First Lady participated in a conference call with the press to announce that she had agreed to be the honorary chair of the Partnership, and that Mayor Cory Booker and Senator Bill Frist would serve as honorary co-chairs. On April 8, 2010, the Board met to approve the appointment of Larry Kocot as interim president and CEO of PHA, the application for Section 501 (c)(3) status and consider a draft strategic plan and commitment process for PHA.

PHA’s board and Founders Committee provided significant expertise on issues related to childhood obesity, and the organization was well positioned to play an independent, non-partisan convener role in facilitating commitments across the private sector. PHA’s first commitment was with the Healthy Weight Commitment Foundation (HWCF), which at the time consisted of 16 of the largest food and beverage companies in the USA. The commitment was the product of discussions between industry, the White House, RWJF and PHA, and was formalized with PHA on May 17, 2010. The HWCF committed to reduce the caloric content of their products by 1.5 trillion calories by 2015. To validate the HWCF commitment, RWJF funded an independent external evaluation that ultimately found that the HWCF exceeded their commitment and reduced 6.4 trillion calories from their products [2]. Moreover, families with children purchased 66 fewer calories per day per person from HWCF brands, a meaningful amount in children’s diets [3].

The commitment process adopted by the board helped establish the operating principles for PHA, including that (1) PHA would work independently, but in support of the *Let’s Move!* initiative; (2) the process to develop and sign commitments with the private sector would reflect meaningful changes in support of honoring good behavior and changing behavior; and (3) PHA would evaluate and publicly report the progress of those commitments.

On May 11, while the HWCF agreement was being negotiated, the White House released the White House Task Force Report on Childhood Obesity. The Taskforce Report outlined key issue areas, including early childhood, empowering parents and caregivers, healthy food in schools, access to healthy, affordable food, and increasing physical activity. Additionally, the report outlined opportunities to create change, including where the private sector could make a difference. Although PHA’s work went beyond the focus areas outlined in the White House Taskforce Report for Childhood Obesity, PHA used the report to establish priorities for potential commitments. Early commitments including the HWCF, early childhood education, WalMart Stores Inc.’s (Walmart) agreement to reformulate foods, and Walmart’s and other retailers’ commitments to build new stores to improve food access, and several commitments that were made later reflect recommendations from the report (Table 2).

**Table 2:** PHA partnerships aligned with the White House Taskforce on Childhood Obesity Report to the President

Goal or target behavior	Strategy	Examples of interventions
Early Childhood	Breastfeeding	Kaiser Foundation Hospitals Inc. commitment to achieve Baby-Friendly USA designation and/or participate in The Joint Commission’s perinatal bundle.
	Early care and education	Bright Horizons, Learning Care Group, KinderCare, and New Horizon Academy commit to provide healthful foods and beverages and to promote physical activity.
Empowering parents and caregivers	Making nutrition information useful	Walmart’s commitment included the “Great For You” food labeling initiative and icon that designates items that meet nutrition criteria informed by the 2010 Dietary Guidelines for Americans. Darden Restaurants Inc., Hyatt Corporation, Subway, Sodexo, and Westin Hotels all committed to improving the nutrition of their children’s meals.
	Food marketing	Sesame and Produce Marketing Associations committed to collaborate to help promote fresh fruits and vegetable consumption to kids. Drink Up marketing campaign promotes water consumption and FNV marketing campaign promotes fruit and vegetable consumption.
	Health care services	700+ hospitals—representing about 10% of all hospitals in the USA—have joined PHA’s Hospital Healthier Food Initiative, providing healthier options for hospital employees and visitors.
Healthy food in schools	Food-related factors in the school environment	Sodexo committed to implement Smarter Lunchroom tactics and serving an additional 17 million free breakfasts in primary and secondary schools. The Mushroom Council committed to pilot recipes for mushroom blend burgers in schools, and in collaboration with Sodexo, 250 school districts across the country will be switching from all-beef burgers for K–12 students to a mushroom-beef blend burger. The Y-USA, Boys & Girls Clubs of America (BGCA) and the National Recreation and Park Association (NRPA) committed to meet nutrition guidelines based on the National AfterSchool Association’s Standards for Healthy Eating and Physical Activity (HEPA) in out-of-school time programs.
Access to healthy affordable food	Physical access to healthy food	PHA’s 7 retail partners, including Walmart, collectively built or renovated 800 locations in areas with low food access, increasing the accessibility of healthier food for more than 8.1 million people across the country. PHA partners include over 1100 convenience store locations across the country, 73% of which are located in food deserts, are expanding healthier food options in their stores. Through PHA’s Healthier Campus Initiative, 44 college and university partners collectively provide healthier food options for more than one million students, faculty and staff across 26 states.
	Food pricing	During the course of its commitment, Walmart saved customers more than $6 billion on fresh produce and had over 470 healthier products that it ensured were priced less than or equal to the less healthy alternative.
	Product formulation	Sixteen major food companies—including Walmart, Sodexo, Subway, Dannon, and Mars Food—have joined in the effort to transform the marketplace, creating healthier options and reducing over 6 trillion calories in the US food supply.
Increasing physical activity	Expanded day and afterschool activities	The Y-USA, BGCA and NRPA committed to meet physical activity standards based on the National AfterSchool Association’s Standards for HEPA in Out-of-School Time Programs.
	The “Built Environment”	Kaiser Foundation Health Plan committed to support Fire Up Your Feet, a youth physical activity program that encourages active transportation to and from school. Four housing developers committed to incorporate active design strategies into their buildings, creating healthier environments for 4650 units of affordable housing across the country
	Community recreation venues	U.S. Tennis Association committed to create at least 5000 kid-sized tennis courts.

Since 2010, PHA has partnered with over 200 companies to increase the supply of healthier options and launched two marketing campaigns to build demand for those options: “Drink Up” to encourage water consumption and “FNV” to encourage the consumption of fruits and vegetables. PHA’s effort to transform the marketplace, as a part of a broader movement to ensure that every child has the chance to grow up at a healthy weight, has become a key component in a multi-sector effort to return child obesity rates to 1970 levels.

### PHA’s Partnership Principles

Market transformation occurs when public health and business sectors work together on agreements that are in the interests of public health and the business interests of the company. This perspective is consistent with CDC’s Guidelines for Public-Private Partnerships [4•] as well as Kraak and Story’s review [5•] of similar partnerships related to the food environment. Both note the importance of an agreement that includes clear goals that benefit both parties and holds partners accountable for outputs or outcomes. The agreement with the HWCF established several key operating principles that have been expanded as PHA’s work has grown.

The first principle is that companies should *stay in their lane*, i.e., a company’s commitment should relate to its core business. For example, a food or beverage company should commit to reduce calories in its products rather than invest in a physical activity intervention.

Partners should make *evidence based*, *meaningful commitments* that address nutrition and physical activity. To ensure that commitments are meaningful, PHA develops commitments with input from a variety of stakeholders. For example**,** public health experts ensure that commitments are informed by current science. At the same time, leaders in the sector help inform PHA about the current landscape and challenges. The most successful commitments balance the ideal with what is feasible. While PHA seeks meaningful commitments, commitments that do not advance the business interests of the company making the commitment are not typically sustainable. Sometimes, the consequence is that commitments may not be transformative (e.g., a company agrees to end production and sales of a sugar beverage), but rather incremental and meaningful (e.g. a company reformulates a sugar drink to reduce calories by 30%). Additionally, PHA considers the potential for initial commitments to create sector-wide changes. For example, PHA’s work with Kwik Trip, Inc. (Kwik Trip), a convenience store chain, led to additional commitments from other convenience store chains and some of the largest distributors in the sector. A subcommittee of PHA’s Board reviews and approves each commitment, and assesses the impact of the commitment.

PHA considers how each *commitment specifically addresses disparities related to obesity*. Disparities became an early PHA focus. However, as PHA grew and the work evolved, consideration of the effect of the commitment on disparities became an element of the approval process. For some larger companies, the impact of a commitment on disparities is often likely but difficult to measure. Nevertheless, PHA tries to ensure that the populations that need change the most will benefit.

PHA commitments require a *signed agreement for each commitment*. PHA distinguishes itself by insisting that partners do more than publicly declare the actions they plan to take. Companies are held accountable through agreed upon metrics, signed contracts, and transparent reporting on progress. At the outset, it is made clear that partners will be held accountable for meeting their commitment, despite leadership changes or economic uncertainty, through a signed agreement that specifies metrics and the timeframe. Progress is assessed through *verification of the commitments* through unbiased, independent third-party evaluators that monitor and publicly report on the progress as part of PHA’s Annual Progress Report.

### PHA Partnerships 2010–2016

The wide range of partners and commitments makes it difficult to quantify the full reach and health impact of all of PHA’s commitments. In addition, we are unable to routinely cite sales data, which would reflect impact, because they are proprietary. Nonetheless, estimates of the reach and outcomes of a number of the commitments of PHA’s partners are summarized below. These estimates reflect the number of individuals or organizations affected by the commitment at the time the commitment was made.

- Seven early childcare companies and out-of-school time providers committed to collectively create healthier environments for more than 6 million children.
- Seven retailers collectively built or renovated 800 locations in areas with low food access, increasing the accessibility of healthier food for more than 8.1 million people across the country.
- Sixteen major food companies—including Walmart, Sodexo Operations, LLC (Sodexo), Subway ®, The Dannon Company, Inc. (Dannon), and Mars Food US, LLC (Mars)—joined an effort to transform the marketplace, creating healthier options and reducing over 6 trillion calories in the US food supply.
- 700+ hospitals—representing about 10% of all hospitals in the USA—joined PHA’s Hospital Healthier Food Initiative, providing healthier options for hospital employees, visitors, and millions of patients.
- Forty-four diverse colleges and universities across 30 states joined PHA’s Healthier Campus Initiative to create healthier campuses for over 1.2 million students, faculty and staff.
- Five major brands—Nike USA, Inc. (Nike), Reebok International LTD., Mercedes-Benz USA, LLC, Dick’s Sporting Goods, Inc., and The North Face, Inc.—committed to invest more than $115.5 million in activities to increase physical activity in US children.
- Four housing developers committed to incorporate active design strategies into their buildings, creating healthier environments for 4000 units of housing across the country.
- Over 1100 convenience stores across the country are expanding healthier food options in their stores, 73% of which are located in food deserts.

#### Company-Level Commitments

PHA partner commitments often result from one-on-one negotiations between PHA and the respective partner. These commitments can represent a core business change, such as reformulation of products or menu changes, or an investment in healthier choices, such as physical activity programming. Examples include Walmart, Nike, Produce Marketing Association (PMA), Sesame Workshop (Sesame), and Dannon.

In January 2012, Walmart, the world’s largest retailer, committed to work with suppliers to reformulate certain packaged food items by reducing sodium by 25% and added sugars by 10%, removing all remaining industrially produced trans fats, assuring that healthier choices are not more expensive than their less healthy counterparts, and developing criteria for a simple front-of-package seal to help customers identify healthier food options. As of December 2015, Walmart had reduced overall sodium by 18% and added sugar by more than 10%. During the course of its commitment, Walmart saved customers more than $6 billion on fresh produce and ensured that over 470 healthier products were priced at less than or equal to the less healthy alternative [6]. Because nearly one third of the US population shops at Walmart each week, Walmart’s commitment to reformulate their products and make healthier products more affordable will likely have a sizable impact on low-income populations.

In February 2013, Nike committed to invest $50 million over 5 years to help create active schools and physically active communities around the US. Nike’s action spurred investments in physical activity among other leading companies. More specifically, Nike has contributed $8.3 million to the collective impact effort of Let’s Move Active Schools, a public-private partnership established in 2013 between the Alliance for a Healthier Generation, the Partnership for a Healthier America, the President’s Council on Fitness, Sports & Nutrition, and SHAPE America, To date, Let’s Move Active Schools represents the collective work of 43 partners and reaches over 20,000 schools and 11 million children across the USA.

The “Eat Brighter!” campaign resulted from a joint commitment with PMA and Sesame Workshop from October 2013. The commitment allows PMA’s community of growers, suppliers, and retailers to use the Sesame Street characters without paying a licensing fee. The campaign produced an impressive 3% increase in produce sales for products that carried Sesame Street characters, and its success led to an extension of the free licensing of Sesame Street characters through 2018 [7].

In March 2014, Dannon, the nation’s leading producer of yogurt, committed to improving nutrient density of its yogurt products, reducing sugar so that 70% of its products by volume contains less than 23 g of sugar per 170 g (6 oz), a standard set by the Institute of Medicine, and reducing fat so that 75% of its yogurt by volume meets FDA standards for “fat-free” or “low in fat.” Dannon’s commitment also included an investment in education and research focused on healthy eating habits. By August 2016, 78% of Dannon’s volume of products and 100% of its children’s products met the standard for sugar [8]. Products meeting the standards have significantly increased from baseline, where 29.2% of children’s products met the standard. Additionally, the number of products that now meet FDA standards for “fat-free” or “low in fat” has increased from 68 to 87% since 2013. On average, the saturated fat and sugar content per 5.3-oz. cup has decreased by 11 and 15%, respectively, over the 3-year commitment.

#### Sector-Wide Commitments

While some commitments result from one-on-one negotiation, others result from a sector-specific commitment with PHA. Sectors have included hospitals, early childhood education providers, convenience stores, colleges and universities, and affordable housing developers.

In May 2011, Bright Horizons Family Solutions LLC (Bright Horizons) was the first company to make a commitment to ensure compliance with a set of guidelines that affected many areas of its early care and education centers. Guidelines included eliminating sugar-sweetened beverages, increasing fruits and vegetables served at every meal, eliminating fried foods, confirming that no time is being spent in front of televisions or video games, limiting computer use to educational activities, and ensuring that children engage in at least 1–2 h of physical activity daily. Later that year, the YMCA of the USA (Y-USA) made a similar commitment, followed 2 years later by KinderCare Learning Centers LLC (KinderCare) and Learning Care Group, Inc. (Learning Care Group). As a result, these partners reach over 1 million children in early childcare settings.

Kwik Trip’s commitment provides another example where PHA was able to develop a sector-wide initiative based on a commitment made by a leading company in that business. In March 2014, Kwik Trip became PHA’s first partner in the convenience store industry when it committed to increase the number of healthier options within its stores, increase the marketing support for these options, and ensure that these options were affordable. Partners now include more than 1000 convenience store locations, and 73% of those are located in areas with lower access to healthful foods. In the first year of its commitment, Kwik Trip increased bulk produce sales by 5.5%, demonstrating the financial benefit from offering healthier options.

New construction of affordable housing offers opportunities to design developments in ways that support physical activity and healthy eating. PHA collaborated with the Center for Active Design and leading experts in the built environment to develop a commitment framework with active design strategies for housing developers. As noted above, PHA has established commitments with four affordable housing developers. These commitments impact over 4000 units, and represent an initial effort to build affordable housing environments that promote health equity and improve health outcomes through active design.

#### Signature Marketing Campaigns

The guiding principles for PHA’s partner work were designed to change the availability of healthier choices. A more recent strategy is the development of campaigns to drive consumer demand. PHA’s two signature marketing campaigns—Drink Up and FNV—focus on engaging, clever, and culturally relevant messages designed to foster an emotional connection with water and fruits and vegetables.

PHA’s Drink Up marketing campaign seeks to increase American’s consumption of water. The Drink Up campaign promotes all water—bottled, filtered, and tap—and is supported by more than 60 companies across every part of the industry. Nielsen Catalina Solutions sales data has demonstrated a 5% increase in water sales in 2015 among those exposed to the Drink Up campaign compared to those not exposed. Data from other national panels, such as the NPD Group, have shown concurrent increases in sales and consumption of water, including in restaurants.

Studies have shown that children are more likely to choose foods endorsed by celebrities, even if that product is a fruit or vegetable [9, 10]. Unfortunately, the majority of food and beverage products currently endorsed by celebrities and athletes are calorie dense and nutrient poor. To shift attitudes towards fruits and vegetables, the Fruit N’ Vegetable (FNV) campaign employs the creative and celebrity star power that other companies have used for decades to sell products. To date, PHA has engaged 85 high-profile celebrities, including NBA MVP Stephen Curry and actress Jessica Alba in advertising, social media, and at events. In its first year, the FNV campaign focused on two lead markets, Virginia Beach and Fresno. Data from the National Marketing Institute found that over a 6-month period in Spring 2016, fruit and vegetable consumption increased or remained constant for 91% of 18–32-year olds in FNV’s two target markets, and 70% of those aware of FNV reported that they consumed more fruits and vegetables after seeing the campaign.

### Lessons Learned

Most discussions with companies do not result in a partnership, in part because PHA’s guiding principles for commitments are rigorous and hold companies publicly accountable. For every new partner announcement made, many others have not come to fruition. Those that do not move forward often reflect the extensive work necessary for a partner to develop a meaningful commitment. Furthermore, some companies did not agree to have their commitment be publicly verified.

Developing private sector commitments that reach the communities whose members face barriers to a healthy weight, such as communities of color, can be more challenging than developing commitments that reach a broader audience. Companies are not always focused on minority or disadvantaged populations. PHA’s efforts often helped them see the potential return on these investments.

One unanticipated result of PHA’s partnerships is that partners have sought ongoing engagement with PHA for nutrition expertise, communications support, or engagement with other partners. In 2015, PHA went through a major reorganization to create a partner relations team to meet these needs.

Developing and implementing commitments are challenging. When developing a commitment, PHA strives to find the balance between the perfect and the feasible. For example, PHA’s Hospital Healthier Food Initiative (HHFI) required hospitals and food service providers to improve the nutritional content of patient meals as well as food options in on-site cafeterias. PHA knew that hospitals had substantial buying power, and that shifts in hospital offerings could also lead to healthier products in the marketplace. However, HHFI required all hospitals to make a variety of changes including offering daily wellness meals and ensuring that 60% of all menu items in the cafeteria and on the patient menu met certain nutrient and food profiles. These guidelines were often difficult to implement. Some lacked the nutrition software to calculate nutrient information. For others, the annual verification required for 3 years became burdensome, especially when hospitals lacked a full list of menu items that included nutrient information and the means to create that list.

When hospitals were able to implement their commitments, they saw positive results. For example, the Henry Ford Health System found that adopting the PHA guidelines improved nutrition choices and also raised cafeteria sales from 2 to 10% depending on the site. Two years after implementing the commitment across its 10 hospitals, MaineHealth’s employee Health Risk Assessment data showed that consumption of five or more fruit/vegetable servings increased from 35% in 2012 to 53% percent in 2014 [11].

The hospital experience informed PHA’s Healthier Campus Initiative (HCI). Each college or university partner is required to meet 23 of 41 established guidelines around nutrition, physical activity, and health and wellness programming on campus. Campuses also have 3 years to implement their changes, but are only required to report on each guideline once. This approach provides more flexibility to implement the guidelines that make the most sense for each campus and reduces the administrative burden of verification. In 2 years since HCI was launched in 2014, PHA has secured 44 partners that collectively reach more than one million students, faculty, and staff across 30 states.

Adherence to a commitment can be especially difficult when businesses face unexpected challenges. Walgreen Co. (Walgreen), one of PHA’s first partners, committed to increase access to fruits and vegetables in at least 1000 stores in or around food deserts. However, the company encountered distribution challenges in getting fresh produce into its stores. Ultimately, Walgreen reported that these challenges prevented them from meeting the commitment, but they continued to add fruits and vegetables to stores where possible. By July 2015, 300 Walgreen stores located in or around food deserts offered fruits and vegetables.

The Y-USA also faced more challenges than anticipated. In November 2011, the Y-USA committed to adopt and implement specific policies and procedures governing healthy eating, physical activity, and screen-time practices in its early childhood and afterschool programs [12–14]. The Y-USA challenge was getting the 728 local YMCA member associations to also pledge to meet standards. By the 2015, 93% of the local Ys had made that commitment. After 4 years, only 40 program sites met 100% of the Healthy Eating and Physical Activity standards and 223 sites met at least 60% of the standards. To help their local Ys implement the standards, Y-USA created additional technical assistance, developed resources and financial incentives, increased healthier vendor options, created workshops, and disseminated best practices.

These examples illustrate efforts to make positive changes, the challenges of implementing systemic changes, a willingness to be held accountable, and the progress that can be made despite falling short of the full commitment.

## Conclusion

One of PHA’s intended impacts is to drive industry to offer and promote healthier options. More companies are now making public commitments on their own, suggesting that a cultural change is underway. Although determining the reach and health impact of private sector commitments is challenging, PHA evaluations demonstrate that changes are occurring in nutrition and physical activity environments. To sustain momentum, PHA will continue to work to foster progress in both supply and demand. The guiding principles that led to over 200 partnerships will continue to guide PHA’s work and provides a model for others. As First Lady Michelle Obama has often noted, change does not occur overnight. Consistent with PHA’s long-term vision, partnerships with the private sector and the efforts of academics, nonprofits, and advocates can help promote health and reduce childhood obesity.
